# Supplementary material for: Microbial Assemblages Associated with the Soil-Root Continuum of an Endangered Plant, Helianthemum songaricum Schrenk
Source: Microbiol Spectr. 2023 May 24;11(3):e03389-22. doi: 10.1128/spectrum.03389-22 (PMC10269481; doi:10.1128/spectrum.03389-22)
Supplement: Supplemental file 1 — Supplemental material. Download spectrum.03389-22-s0001.pdf, PDF file, 1.9 MB [file spectrum.03389-22-s0001.pdf]

Supplementary Materials

Microbial assemblages associated with the soil-root continuum of endangered plants, *Helianthemum songaricum* Schrenk

Daolong Xu[a], Xiaowen Yu[b], Jin Chen[c], Haijing Liu[a], Yaxin Zheng[a], Hanqing Qu[a], Yuying Bao[a]*

a. Inner Mongolia Univ, Sch Life Sci, Minist Educ, Key Lab Forage & Endem Crop Biotechnol, Hohhot 010021, Peoples R China.

b. Inner Mongolia Autonomous Region Environmental Monitoring Station, Hohhot, 010021, China.

c. National Engineering Laboratory of Crop Stress Resistance Breeding, Anhui Agricultural University, Hefei 230036, Peoples R China.

*Corresponding author Address:Inner Mongolia Univ,235 West Univ Rd,Hohhot 010021,Inner Mongolia,Peoples R China.

Tel/Fax:+86 471 4492944

E-mail address:ndbyy@imu.edu.cn

Supporting Information Includes:

2 Table

3 Figures

Captions:

Table S1 Basic characteristics of the *Helianthemum songaricum* Schrenk site[a].

Table S2. Alpha-diversity indices of *Helianthemum songaricum* Schrenk samples.

Fig.S1. Bacterial and fungal venn diagrams(a,b)in rhizosphere soils and roots sample of *Helianthemum songaricum* Schrenk at the Class levels

Fig.S2. Rarefaction curve analysis. The curve was constructed using the Shannon index of Class and the number of reads.

Fig.S3. Composition of microbial communities in rhizosphere soils and roots sample of *Helianthemum songaricum* Schrenk at the Class levels. a,b:Bacterial community. c,d: Fungi community.

Table S1 Basic characteristics of the *Helianthemum songaricum* Schrenk site[a].

Sites	Site code[a]	Location	Soil Type	Altitude (m)	Plant type	pH	Soil TN(g/kg)	SOM (g/kg)	Soil TP (mg/kg)
Ordos City (Qipanjing)	S-BRH	N:39°22′03″E :107°01′48″	Chestnut soil andbrown soil	1342	Shrub and Herbs	8.16±0.17a	0.189±0.032a	7.212±1.0664a	0.188±0.014a

Table S2. Alpha-diversity indices of *Helianthemum songaricum* Schrenk samples.

Sample code	Target	Final reads	OTUs	Chao1	Goods Coverage	Shannon	Average Length(bp)
S-BRH	16Sr DNA	163139	2118	65	0.9996	2.016	414.953
	ITS	60974	553	7	0.9999	0.626	257.754
R-BRH	16Sr DNA	22935	314	58.964	0.9998	2.262	406.439
	ITS	26919	65	5.667	0.9999	0.959	269.482

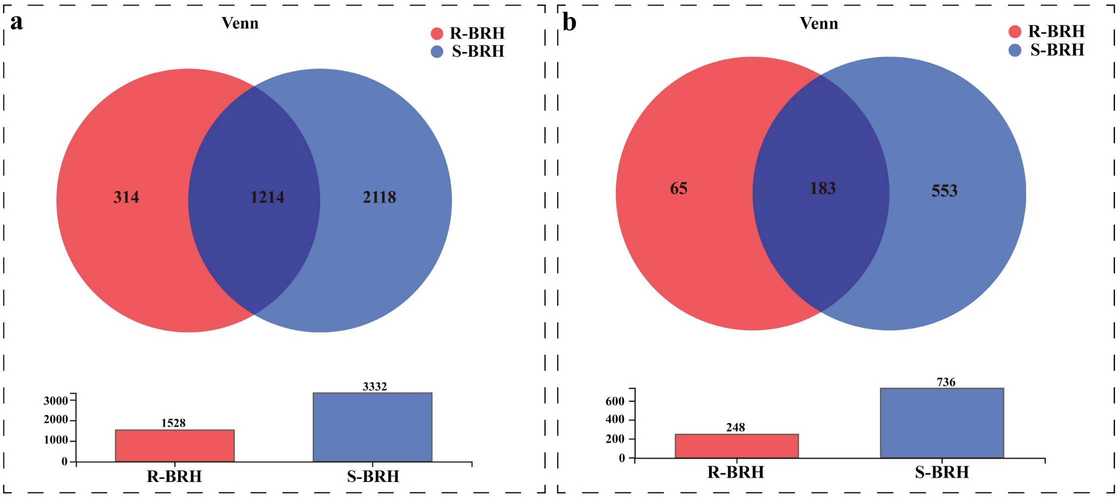

Fig.S1. Bacterial and fungal venn diagrams(a,b)in rhizosphere soils and roots sample of *Helianthemum songaricum* Schrenk at the Class levels

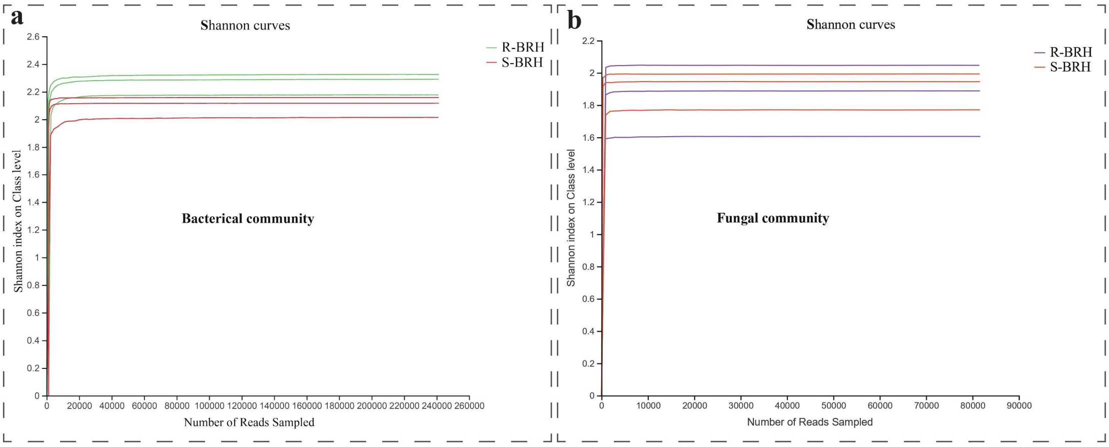

Fig.S2. Rarefaction curve analysis. The curve was constructed using the Shannon index of Class and the number of reads.

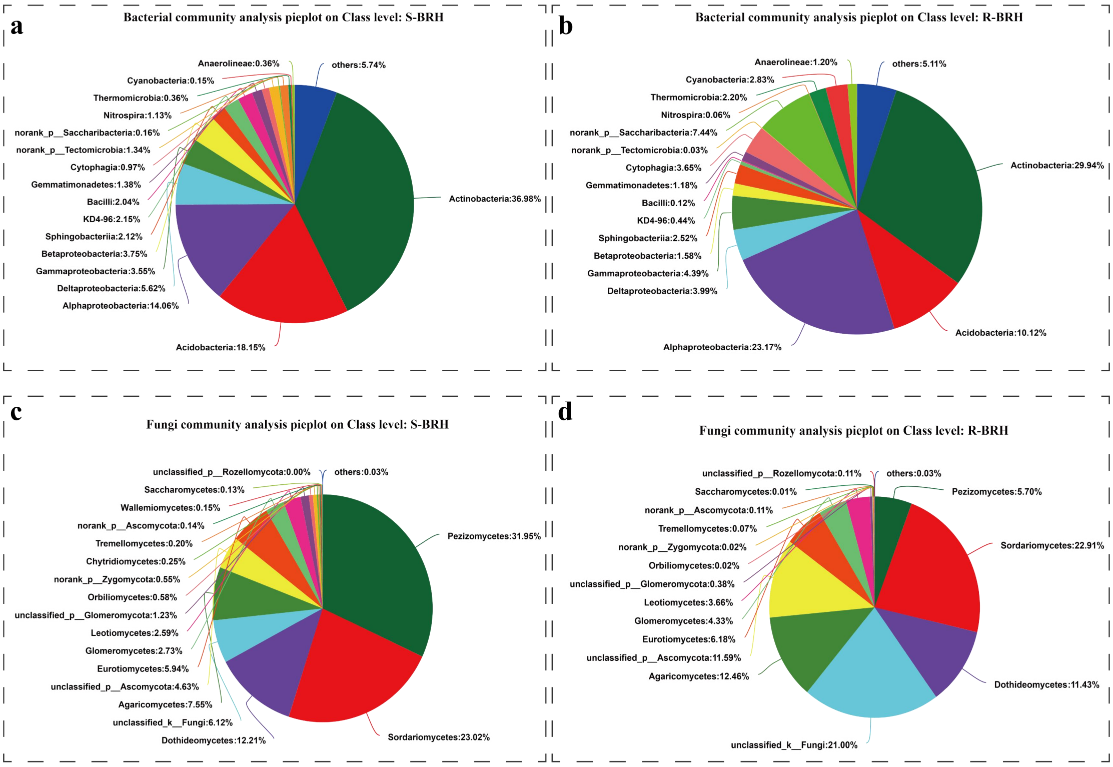

Fig.S3. Composition of microbial communities in rhizosphere soils and roots sample of *Helianthemum songaricum Schrenk* at the Class levels.a,b:Bacterial community.c,d:Fungi community.
